# *Enterobacter cloacae* from urinary tract infections: frequency, protein analysis, and antimicrobial resistance

**DOI:** 10.1186/s13568-024-01675-7

**Published:** 2024-02-08

**Authors:** Ayman Elbehiry, Mansor Al Shoaibi, Hamzah Alzahrani, Mai Ibrahem, Ihab Moussa, Feras Alzaben, Rousa A. Alsubki, Hassan A. Hemeg, Dakheel Almutairi, Saleh Althobaiti, Fawaz Alanazi, Sultan A. Alotaibi, Hamoud Almutairi, Ali Alzahrani, Akram Abu-Okail

**Affiliations:** 1 Department of Public Health, College of Public Health and Health Informatics, Qassim University, 52741 Al Bukayriyah, Saudi Arabia; 2 Department of Support Service, King Fahad Armed Hospital, 23311 Jeddah, Saudi Arabia; 3 Department of Preventive Medicine, King Fahad Armed Hospital, 23311 Jeddah, Saudi Arabia; 4 Department of Public Health, College of Applied Medical Science, King Khalid University, 61421 Abha, Saudi Arabia; 5 Department of Botany and Microbiology, College of Science, King Saud University, 11451 Riyadh, Saudi Arabia; 6 Department of Food Service, King Fahad Armed Forces Hospital, 23311 Jeddah, Saudi Arabia; 7 Department of Clinical Laboratory Science, College of Applied Science, King Saud University, Riyadh, Saudi Arabia; 8 Department of Medical Laboratory Technology, College of Applied Medical Sciences, Taibah University, Madinah, Saudi Arabia; 9 Medical Transportation Administration of Prince Sultan Military Medical City, 12233 Riyadh, Saudi Arabia; 10 Pharmacy Department, Armed Forces Hospital in Jubail, 35517 Jubail, Saudi Arabia; 11 Supply Administration, Armed Forces Hospital, King Abdul Aziz Naval Base in Jubail, 35517 Jubail, Saudi Arabia; 12 Medical Administration, Armed Forces Hospital, King Abdul Aziz Naval Base in Jubail, 35517 Jubail, Saudi Arabia; 13 Aviation Medicine, King Abdulaziz Medical City of National Guard, 14611 Riyadh, Saudi Arabia; 14 Department of Veterinary Medicine, College of Agriculture and Veterinary Medicine, Qassim University, 52571 Buraydah, Saudi Arabia

**Keywords:** *Enterococcus cloacae*, Identification, Antibiotic agent, Public health

## Abstract

The genus Enterobacter belongs to the ESKAPE group, which includes *Enterococcus faecium*, *Staphylococcus aureus*, *Klebsiella pneumoniae*, *Acinetobacter baumannii*, *Pseudomonas aeruginosa*, and *Enterobacter spp*. This group is characterized by the development of resistance to various antibiotics. In recent years, *Enterobacter cloacae* (*E. cloacae*) has emerged as a clinically important pathogen responsible for a wide range of healthcare-associated illnesses. Identifying *Enterobacter* species can be challenging due to their similar phenotypic characteristics. The emergence of multidrug-resistant *E. cloacae* is also a significant problem in healthcare settings. Therefore, our study aimed to identify and differentiate *E. cloacae* using Matrix-assisted laser desorption ionization–time of flight mass spectrometry (MALDI-TOF MS) as a fast and precise proteomic analytical technique. We also tested hospital-acquired *E. cloacae* isolates that produce Extended-spectrum beta-lactamases (ESBL) against commonly used antibiotics for treating urinary tract infections (UTIs). We used a total of 189 *E. cloacae* isolates from 2300 urine samples of patients with UTIs in our investigation. We employed culturing techniques, as well as the BD Phoenix™ automated identification system (Becton, Dickinson) and Analytical Profile Index (API) system for the biochemical identification of *E. cloacae* isolates. We used the MALDI Biotyper (MBT) device for peptide mass fingerprinting analysis of all isolates. We utilized the single peak intensities and Principal Component Analysis (PCA) created by MBT Compass software to discriminate and cluster the *E. cloacae* isolates. Additionally, we evaluated the sensitivity and resistance of ESBL-*E. cloacae* isolates using the Kirby Bauer method. Out of the 189 *E. cloacae* isolates, the BD Phoenix system correctly identified 180 (95.24%) isolates, while the API system correctly identified 165 (87.30%) isolates. However, the MBT accurately identified 185 (98.95%) isolates with a score of 2.00 or higher. PCA positively discriminated the identified *E. cloacae* isolates into one group, and prominent peaks were noticed between 4230 mass-to-charge ratio (m/z) and 8500 m/z. The ESBL-*E. cloacae* isolates exhibited a higher degree of resistance to ampicillin, amoxicillin-clavulanate, cephalothin, cefuroxime, and cefoxitin. Several isolates were susceptible to carbapenems (meropenem, imipenem, and ertapenem); however, potential future resistance against carbapenems should be taken into consideration. In conclusion, MALDI-TOF MS is a powerful and precise technology that can be routinely used to recognize and differentiate various pathogens in clinical samples. Additionally, the growing antimicrobial resistance of this bacterium may pose a significant risk to human health.

## Introduction

Globally, urinary tract infections (UTIs) are one of the major and expensive healthcare problems worldwide due to their high frequency, treatment challenges, and negative consequences (Öztürk and Murt 2020; Dehkordi et al. 2022; Mekonnen et al. 2023). They can impact individuals of all ages, from children to adults. The structure of the female genital system, specifically the close proximity of the urine meatus to the anus, makes women more susceptible to UTIs compared to men (John et al. 2016; Chen et al. 2023). Approximately 250 million people are affected by UTIs every year, accounting for roughly 40% of all infections worldwide and resulting in a global economic burden exceeding $6 billion (Tan and Chlebicki 2016; Delcaru et al. 2017; Gajdács and Urbán, 2019). UTIs are associated with a significant burden of mortality and a wide range of clinical signs, from asymptomatic bacteriuria to cystitis or septic shock, which may result in dangerous multi-organ dysfunction (Agarwal et al. 2012; Singh et al. 2017; Ratajczak et al. 2022).

The *Enterobacter* species belong to the *Enterobacteriaceae* family, which is a group of gram-negative facultative anaerobic bacteria. As a member of the human alimentary tract's commensal flora, these bacteria are also classified as plant, insect, and human pathogens (Mezzatesta et al. 2012). A wide variety of *Enterobacter* spp. can be found throughout the world and can be collected from outdoor environments, animals, and healthcare settings (Davin-Regli et al. 2019; Ganbold et al. 2023; Ortiz-Díez et al. 2023). As one of the ESKAPE groups of bacteria, *Enterobacter* spp. play a significant role in poorer health outcomes and increased therapy costs (De Oliveira et al. 2020; Catalano et al. 2023). These bacteria are responsible for a variety of nosocomial and community-acquired illnesses, including infections of the urinary system, lungs, and soft tissues (Patel and Patel 2016; Davin-Regli et al. 2019; Anju et al. 2020). Various types of microorganisms are responsible for most UTIs, with *Enterobacteriaceae* being the most prevalent (Pardeshi 2018; Azar et al. 2023; Mancuso et al. 2023b). The most prevalent *Enterobacteriaceae* are *Escherichia coli*, followed by *Klebsiella pneumoniae* and *E. cloacae* (Toner et al. 2016; Takei et al. 2023).

There is a clinical significance associated with the *E. cloacae* complex group (Candela et al. 2023). According to the sequencing of the Heat Shock Protein 60 (*hsp60*) genome, this group consists of 13 diverse genetic clusters. However, the classification of this genus is still in question (Hoffmann and Roggenkamp 2003; Davin-Regli et al. 2019). By using whole-genome sequencing (WGS) data gathered from isolates of the *E. cloacae* complex, the species identified by *hsp60* sequencing (5) were reorganized into distinct classes (Sutton et al. 2018) and new species of the *E. cloacae* complex were discovered (Wu et al. 2019). Most *E. cloacae* complex species are distinguished using sequence-based approaches. The *hsp60* gene is commonly used, but WGS and multilocus sequence typing are also utilized (Hoffmann and Roggenkamp 2003; Singh et al. 2018). Sequence-based testing techniques require a significant amount of time and specialized equipment to be successful. Several new approaches, such as MALDI-TOF MS, are being developed to replace sequence-based methods. The MALDI-TOF MS method has been successfully revealed as a influential technique for identifying bacteria (Elbehiry et al. 2017, 2019b, 2021, 2022a, 2022b, 2023; Al-Beloushi et al. 2018; Abalkhail and Elbehiry 2022). It can recognize *E. cloacae* complex isolates at the species level, but its differentiation ability for these species is limited when conventional methods are conducted and commercially available databases with poor determination are used (Pavlovic et al. 2012; De Florio et al. 2018).

Another significant issue of concern within the *E. cloacae* complex is antibiotic resistance, which is a consequence of the incorrect and inappropriate use of antibiotics (Intra et al. 2023). The *E. cloacae* complex belongs to the ESKAPE group, which is identified as the most common cause of healthcare-associated infections. In recognition of this, the World Health Organization has added *Enterobacter* spp. to the list of priority bacteria for the development of novel antibiotics (Davin-Regli et al. 2019; Intra et al. 2023). Many types of *Enterobacter* spp. have developed resistance to ampicillin and wide-spectrum cephalosporins, as well as other antimicrobial drugs such as third-generation cephalosporins and carbapenems, due to the acquisition of genetic mobile elements. This has made treatment difficult (Annavajhala et al. 2019; Davin-Regli et al. 2019; Quraishi et al. 2019).

Furthermore, *E. cloacae* is inherently resistant to amoxicillin, ampicillin, cefoxitin, and cephalosporins, regardless of whether it produces the *AmpC*-lactamase enzyme. Cephalosporins, which are broad-spectrum antimicrobial drugs, are highly susceptible to enzymatic resistance (Zahra et al. 2021; Rabaan et al. 2022). Excessive production of *AmpC*-lactamase often leads to resistance to third-generation cephalosporins. Treatment with cephalosporins can result in the overproduction of *AmpC* mutants (Cairns et al. 2021). Fourth-generation cephalosporins have limited effectiveness against de-repressed bacteria, but in the presence of ESBL-producing strains, the bacteria can develop resistance to the aforementioned antimicrobials (Ahmed et al. 2022). However, there is currently no vaccine available to protect against *E. cloacae* infections, highlighting the need for the development of a highly effective vaccine to combat these bacterial infections (Ioannou et al. 2022).

Several research studies (Ali et al. 2016; Neupane et al. 2016) have found an increase in antibiotic resistance in *Enterobacteriaceae* strains from UTIs to commonly prescribed antibiotics like ciprofloxacin and trimethoprim-sulfamethoxazole. Both European and African investigations have stated a significant rise in Gram-negative bacteria associated with Extended-spectrum beta-lactamase (ESBL)-producing UTIs (Barguigua et al. 2013; Hammami et al. 2013). The rise of ESBL-generating microorganisms has made empirical therapy of diseases more challenging and has also led to increased resistance to beta-lactams such as penicillin and cephalosporins, and in some cases, carbapenems (Paterson and Bonomo 2005). There is a strong relationship between the development of antibiotic resistance in *Enterobacter* spp. and higher mortality rates, prolonged hospital stays, and higher medical expenditures for those infected (Davin-Regli et al. 2019). There have been a few articles published about the resistance of *Enterobacter* spp. to antibiotics (Wang et al. 2017; Jiménez-Guerra et al. 2020; Liu et al. 2021).

Based on the previously mentioned data, it is crucial to rapidly identify and differentiate *E. cloacae* in order to address the issue of phenotypic similarity among *E. cloacae* complex species. Furthermore, it is necessary to analyze the antibiotic susceptibility profiles of *E. cloacae* in healthcare-associated infections to determine the degree of tolerance and choice the most suitable antibiotic treatment for UTIs. In Saudi Arabia, there has been limited research on the characteristics of ESBL-*E. cloacae* and its antibiotic resistance, so conducting such an investigation could be beneficial for clinicians when choosing an appropriate empirical antibiotic therapy. Therefore, this study focused on utilizing MALDI-TOF MS for proteomic identification and differentiation of *E. cloacae* isolates obtained from UTI patients. Additionally, antibiotic resistance profiles were conducted to identify the most effective antibiotics for therapy.

## Materials and methods

### Ethical approval

Since there were no human participants in this study, ethical approval or written authorization was not required. We only used archived bacterial cultures obtained from routine medical tests. Prior to accessing any data, all cultures were completely anonymized.

### An overview of the study's design and data collection

During January to December 2020, the Microbiology Laboratory at King Fahd Medical City conducted a randomized exploratory investigation. The study focused on isolating all *E. cloacae* isolates from urine specimens. The microbiology laboratory within the hospital provided information on sample collection and bacterial isolation, and the authors did not directly contact the patients for this study. To gather patient information, the clinical microbiology laboratory used its service management program. Following standard laboratory procedures, the clinical samples of the patients were collected and analyzed. Each patient's midstream urine sample was collected and placed in sterile plastic containers. Using a disposable plastic loop, the specimens were streaked on blood agar and MacConkey agar and incubated at 37 °C for 24 h. Initially, a preliminary reading was taken, and then the specimen was further incubated for a final reading. There were three categories of final culture reports: pure, mixed, and no growth. If two organisms were present, they were considered mixed, and in most cases, we couldn’t identify them precisely. For the pure cultures, species and colony-forming units per milliliter were used for further classification. For a urine specimen to be considered to have significant monomicrobic bacteriuria (urine bag), it needed to contain more than 10^5^ CFU/mL of a specific microbial species. Patients with multimicrobic bacteriuria or whose urine samples were taken more than three days after admission were excluded from the study.

In total, 2300 urine samples were included in this study. To ensure accurate estimation of resistance levels, only the first isolate of *E. cloacae* from each participant was used. The isolates were classified based on whether they came from outpatient care or from a hospital setting. Outpatient samples were collected from the hospital's outpatient sections and from physicians in the surrounding community. Hospitalized patients were sampled from the hospital’s emergency room and various hospital units. The collected samples were recorded and submitted to the microbiology lab for additional testing, following contemporary European regulations. If the specimens could not be examined immediately, they were chilled at 4 to 6 °C (Cornaglia et al. 2020).

### Culturing technique

In accordance with the guidelines on routine culture media from the World Health Organization, all urine samples were cultured using a semiquantitative method (Vandepitte 2003). To isolate *E. cloacae* bacteria, one mL of each urine sample was plated on nutritional agar, tryptose soya agar, and two differential selective media, including Xylose Lysine Deoxycholate agar (XLD) and *Salmonella Shigella* agar (SS). The culture plates were then incubated at 37 °C for 18–24 h. The presence of *E. cloacae* was determined by observing the appearance of rough and smooth colonies on tryptose-soya agar. In this study, biochemical assays and cultural features were utilized to identify and classify the bacteria, utilizing the BD Phoenix™ automated identification system (BD—Mississauga, Canada) and Analytical profile index (API) system (bioMerieux UK Ltd, Basingstoke, UK).

### BD Phoenix System-based biochemical identification of E. cloacae isolates

Various types of Gram-negative bacteria were tested using the NMIC/ID-4 panel of the Phoenix system. To prepare the McFarland 0.5 stock solution, 4.5 mL of Phoenix ID broth was used based on the SensititreTM Nephelometer (Thermo Fisher Scientific, Waltham, USA) (Carroll et al. 2006). The obtained bacterial ID solution was then filled into the ID field of the Phoenix panel. A 50 µL solution of bacterial solution was added to each of the chemical reactivity wells on the panel. Any excess suspension was absorbed by the cushion beneath the panels. Once the panel was coated with a rubberized coating and the display code was scanned, it was immediately inserted into the Phoenix machine. To compare results, the ID results of *E. cloacae* isolates from urine samples using the Phoenix system were compared with those of the old API system.

### The identification of *E. cloacae* by MALDI Biotyper (MBT)

According to Lartigue (2013), the MBT was used to identify 186 isolates of *E. cloacae* from various urine samples. The ethanol-formic acid-acetonitrile extraction scheme developed by Bruker Daltonics in Bremen, Germany was used for this purpose. The isolates were streaked on nutrient agar (Hardy Diagnostics, Saudi Arabia) and incubated at 30 °C for 24 h. To minimize randomization, two duplicates of a fresh colony from each isolate were injected onto the MBT 96 target plate. A 1 µL matrix solution consisting of saturated -Cyano-4-hydroxycinnamic acid (CHCA), 50% acetonitrile (MeCN), and 2.5% trifluoroacetic acid (TFA) was mixed with the colony. The Compass software installed in the MBT device was used to identify unknown bacteria (see Table 1).

**Table 1:** An analysis of key factor indicators in urine *E. cloacae* isolates from January to December 2020

Indicator	Isolates of non-ESBL-*E. cloacae* (No = 107)		Isolates of ESBL-*E. cloacae* (No = 82)		Total (No = 189)	
Sex						
Female	59	55.14%	44	53.66%	103	54.50%
Male	48	46.60%	38	46.34%	86	45.50%
Age						
0–10 year	28	26.17%	19	23.17%	47	24.87%
11–20 year	3	2.80%	2	2.44%	5	2.65%
21–30 year	5	4.67%	1	1.22%	6	3.17%
31–40 year	7	6.54%	5	6.10%	12	6.35%
41–50 year	19	17.76%	17	20.73%	36	19.05%
51–60 year	15	14.02%	13	15.85%	28	14.81%
61–70 year	16	14.95%	15	18.29%	31	16.40%
71–80 year	14	13.09%	10	12.20%	24	12.70%

An electric field of 20 kV was used to propel ions in a positive ion mode. Ions with masses greater than 1000 Da could be extracted using the pulse extraction procedure. Spectra were automatically gathered and compared to those of reference strains of *E. cloacae* using the Compass software and Flex control of laser intensity. A spectrum analysis was then performed. Logarithmic scores were calculated by comparing unknown spectra to spectra in the database used as references. A score of 2.0 or higher indicates reliable species identification, while scores ranging from 1.9 to 1.7 are acceptable for genus identification. The MBT spectra were grouped based on correlated distances between them using the Main Spectrum Profile (MSP) dendrogram. The clusters were divided and examined using distance levels of 100, 180, and 500. *Escherichia coli* (*E. coli*) positive control was used as a bacterial test standard during the investigation.

### Antimicrobial resistance of *E. cloacae* isolates using the Kirby-Bauer test

A Kirby Bauer test was used to evaluate the susceptibility and resistance to antibiotics of all isolates of *E. cloacae* that had been identified according to the Clinical Laboratory Standards Institute (CLSI) guidlines for version 6.0 of their guidelines (Kosikowska et al. 2020). An evaluation of 22 antimicrobial agents against *E. cloacae* was conducted (Table 2). An experimental analysis was conducted, and the results are presented as Susceptible/Intermediate/Resistant according to CLSI’s indicated diameters or breakpoints (Kosikowska et al. 2020). Once an isolate is found to be resistant to three different classes of antibiotics, it is classified as multidrug-resistant.

**Table 2:** The antibiotic resistance and susceptibility profiles of ESBL *E. cloace* isolates from urine of patients suffering from UTIs

Antimicrobial drug	Concentration (µg)	Resistant		Susceptible	
		Number of isolates	Percentage (%)	Number of isolates	Percentage (%)
Ampicillin	10	82	100	0	0.00
Amoxicillin–Clavulanate	20/10	82	100	0	0.00
Cephalothin	30	82	100	0	0.00
Cefuroxime	30	66	80.49	16	19.51
Ceftazidime	30	11	13.23	71	86.77
Cefoxitin	30	80	97.56	2	2.44
Cefepime	30	11	13.41	71	86.57
Cefotaxime	30	19	23.17	63	76.83
Ceftriaxone	30	19	23.17	63	76.83
Ciprofloxacin	5	11	13.41	71	86.57
Gentamicin	10	6	7.32	76	92.68
Amikacin	30	0	0.00	82	100
Trimethoprim–Sulfamethoxazole	1.25/23.75	19	23.17	63	76.83
Piperacillin-Tazobactam	36	3	3.66	79	96.34
Imipenem	10	3	3.66	79	96.34
Meropenem	10	3	3.66	79	96.34
Ertapenem	10	8	9.76	74	90.24
Levofloxacin	5	3	3.66	79	96.34
Tigecycline	15	11	13.41	71	86.57
Nitrofurantoin	300	11	13.41	71	86.57
Aztreonam	30	6	7.32	76	92.68

### Disc susceptibility test for detection of ESBL-E. cloacae

The ESBL-*E. cloacae* was detected in all strains using three cephalosporin markers: cefpodoxime (30 g), ceftazidime (30 g), and cefotaxime (30 g). A resistant isolate was identified as one with a zone diameter of 17 mm, 22 mm, and 27 mm for each of the three markers, respectively. A phenotypic confirmation test was performed on isolates that showed resistance to at least one of the three cephalosporins (New 2007).

### Double-disk synergy test (DDST) for ESBL producing E. cloacae

*Enterobacter cloacae* isolates tested by DDST proved to be tolerant to all three antimicrobial agents used for ESBL development. The ESBLs were identified using cefpodoxime (30 g), ceftazidime (30 g), cefotaxime (30 g), and amoxicillin/clavulanic acid (20 g amoxicillin + 10 g clavulanic acid). Discs of amoxicillin/clavulanic acid (20 g/10 g) and third-generation cephalosporin were placed 20 mm apart on Muller-Hinton agar (Hardy Diagnostics, Saudi Arabia) plates. The plates were then incubated overnight at 37 °C. ESBL findings were considered positive if the cephalosporin inhibition zone increased towards the amoxicillin/clavulanic acid disc. DSM 30054 *E. cloacae* subsp. *cloacae* and DSM 498—*E. coli* were used as positive controls.

### Multiple antibiotic resistance (MAR) index determination

MAR index is an evaluation method for measuring the possible dangers and testing for multidrug-resistant microorganisms. In this experiment, 21 antimicrobial agents were tested, which are indicated by the letter (b). As stated by Osundiya et al. (2013), The MAR index is calculated by dividing the number of antimicrobial agents to which the isolate conferred resistance (a) by the total number of antimicrobial agents utilized (b).

### Analysis of statistical data

Data was analyzed using SPSS Software 21.0 (SPSS, Chicago, IL, USA).

## Results

### Biochemical identification of Enterobacter cloacae isolates

After examining 2300 urine samples, 189 isolates of *E. cloacae* were identified using a culture and staining procedure. Out of all the isolates, the BD Phoenix System accurately identified 180 (95.24%) isolates, while misidentifying nine (4.76%) isolates. On the other hand, the API system correctly identified 165 (87.30%) out of the total isolates.

### Proteome identification of Enterobacter cloacae

**Fig. 1:**
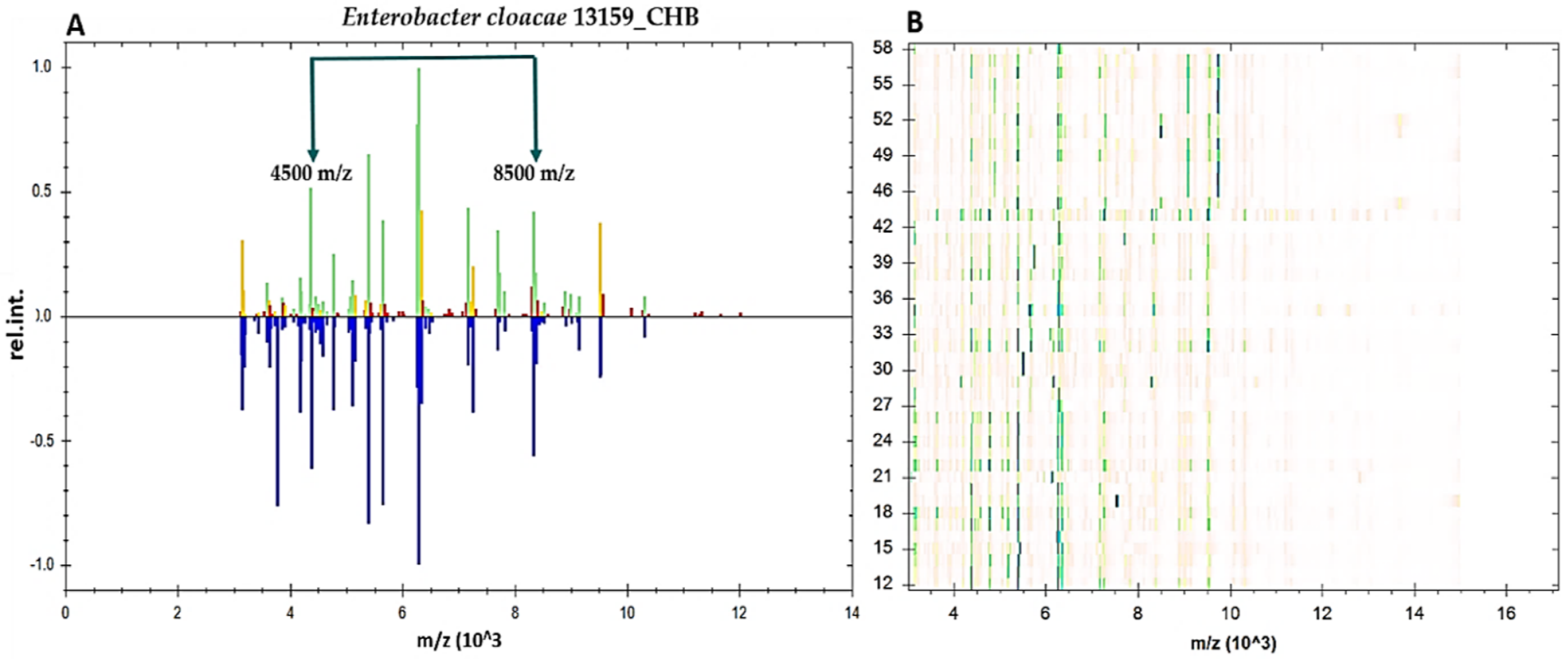
**A** Analysis of the spectral profile of one *E. cloacae* isolated from urine samples, compared to the profile of *E. cloacae* 13159_CHB, a reference strain in the MBT library. The deposited spectrum is shown in blue in the lower half of the spectra, while the green spectrum in the top part reveals prominent matching peaks. Mismatched peaks are shown in red, and intermediate peaks are shown in yellow. The concentrations of peaks observed in line spectra range from 3500 to 10,500 m/z, with prominent peaks occurring between 4230 and 8500 m/z. **B** Gel profiles constructed from various protein spectra of various *E. cloacae* isolates

A total of 189 isolates of *E. cloacae* were analyzed using the MBT automated machinery. The Compass software’s Bruker library was used to compare the acquired spectra, and 185 out of 189 (97.88%) were correctly detected. According to the results, 98 isolates (51.95%) had scores between 2.30 and 3.00, while 82 isolates (43.38%) had scores between 2.00 and 2.29. The genus level identification included five isolates with scores ranging from 1.7 to 1.99. The remaining four isolates were identified as *Klebsiella pneumoniae*. A recent gel photograph depicted the distinct spectra of each isolate of *E. cloacae*. In Fig. 1A, multiple spectra were recorded ranging from 3500 m/z to 10,500 m/z, but the prominent peaks were found between 4230 m/z and 8500 m/z. Our study found that the spectral protein profiles of reference strains from the MBT collection matched the gel views of protein for isolates of *E. cloacae* from urine samples (Fig. 1B).

**Fig. 2:**
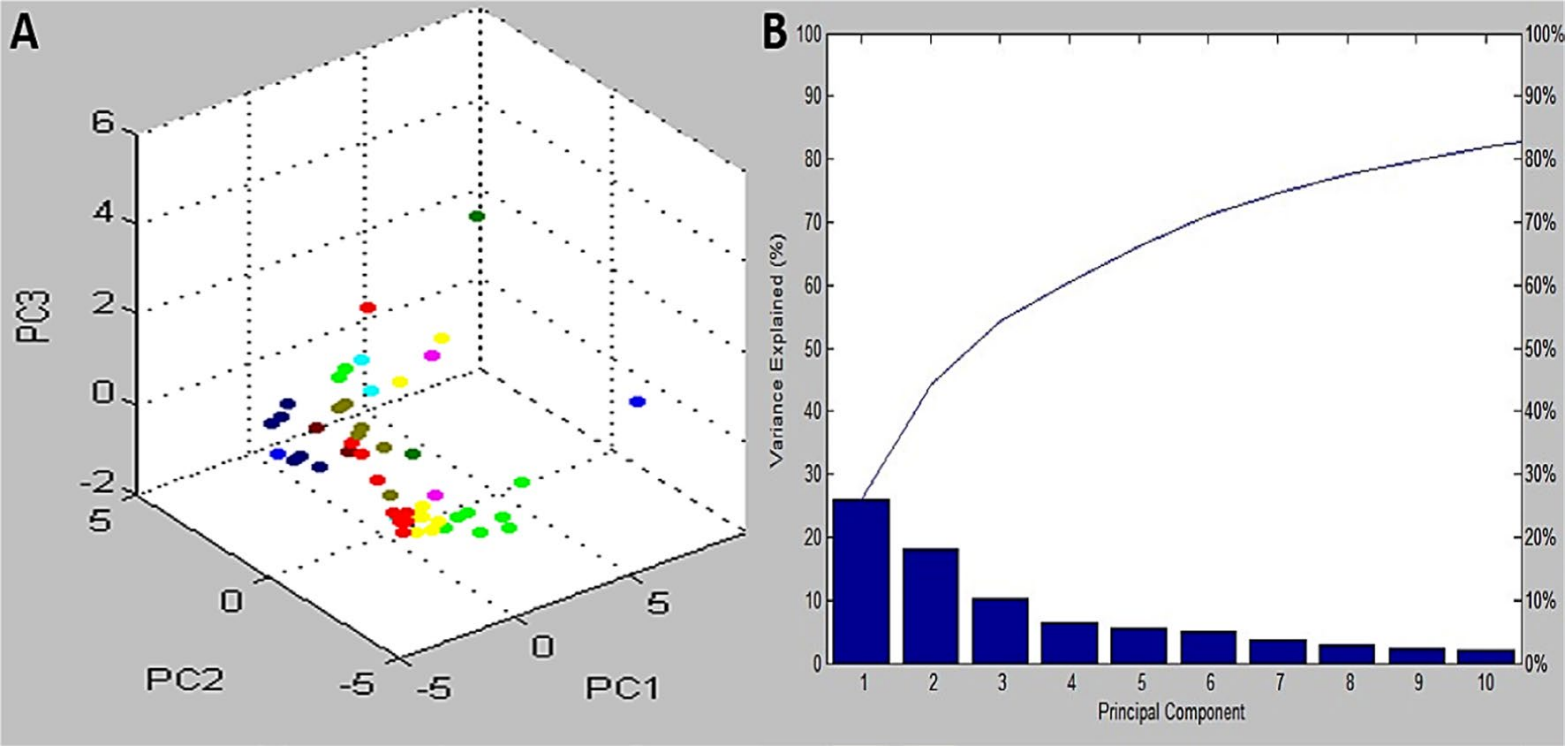
**A** The PCA image displays a three-dimensional image of the field E cloacae isolates. **B** PC plots demonstrate the influence of ten main components on profile categorization. The first three components (PC1, PC2, and PC3) contributed approximately 26%, 18%, and 13% respectively

Additionally, we utilized the PCA feature of the MBT Compass software to evaluate the variation and similarity of protein spectra. As shown in Fig. 2A, the identified isolates exhibited a wide range of spectral signals associated with their proteins. Each peak was determined by employing three loading values. These loading values were calculated based on the three principal component equations (PC1, PC2, and PC3). The PCA tool was employed in our analysis to examine all observed peaks in the MBT database. According to the 3D PCA, all isolates of *E. cloacae* were clustered together. An analysis of the PCA revealed that PC1, PC2, and PC3 had reciprocal impacts on the development of a specific characteristic, accounting for nearly 26%, 18%, and 13% (Fig. 2B), correspondingly.

According to our results, the MSP-based dendrogram showed clear relationships between the six reference strains in the reference library and the tested *E. cloacae* isolates. A total of 189 clinical isolates of *E. cloacae* were matched to the following strains used as references in the study: 65 isolates with *E. cloacae* 13159_CHB, 55 isolates with *E. cloacae* ssp *cloacae* DSM 30054T HAM, 33 isolates with *E. cloacae* 20105_2 CHB, 17 isolates with *E. cloacae* MB 8779 05 THL, 12 isolates with *E. cloacae* DSM 3264 BRB, and 7 strains were found to be matched with *E. cloacae* DSM 3060 DSM.

### Frequency of ESBL and non-ESBL E. cloacae

During the investigation, 2300 urine samples that met the eligibility requirements were collected from patients with UTIs in both outpatient and inpatient settings at King Fahd Medical City. Out of these specimens, 189 (8.21%) contained *E. cloacae* isolates from patients with nonrepetitive UTIs. Among these, 82 (43.39%) contained ESBL-*E. cloacae* and 107 (56.61%) contained non-ESBL-*E. cloacae*. When looking at gender, female patients with ESBL-*E. cloacae* accounted for 53.66% while male patients accounted for 46.34%. For non-ESBL-*E. cloacae*, female patients accounted for 55.14% and male patients accounted for 46.6%. These findings suggest that the spectrum of *E. cloacae* differs significantly between male and female UTI patients, with females being more likely to develop the infection. In terms of age, patients aged 0–10 years had the highest percentage (24.87%) of both ESBL and non-ESBL *E. cloacae*. This was followed by patients aged 41–50 years (19.05%), 61–70 years (16.40%), 51–60 years (14.81%), and 71–80 years (12.70%).

### The degree of resistance and susceptibility of ESBL E. cloacae to various antibiotic classes

**Fig. 3:**
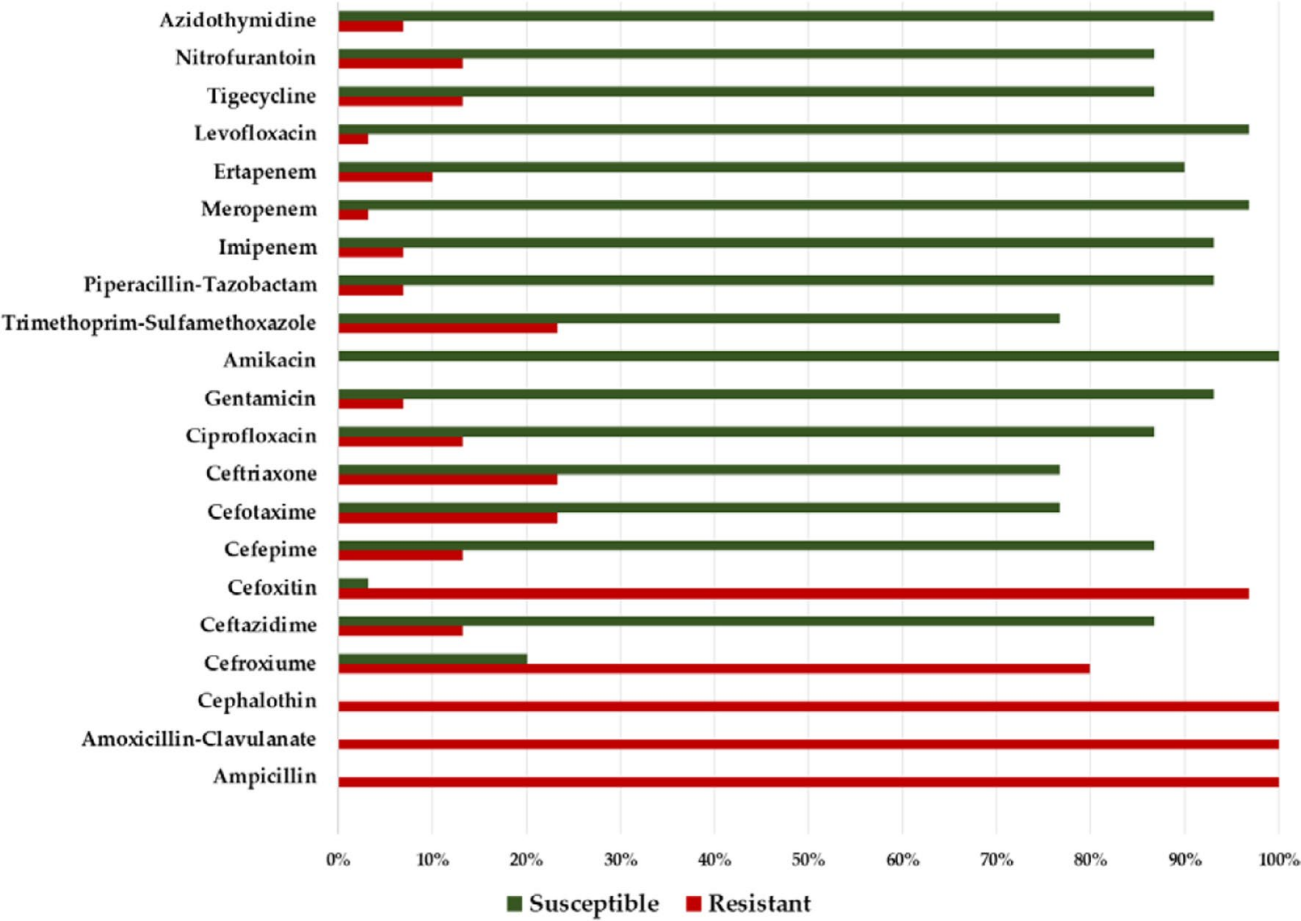
Antimicrobial resistance and susceptibility patterns of 82 ESBL *E. cloacae strains*

Our analysis of the data in Table 2 included six classes of 21 antimicrobial agents. All isolates of ESBL-*E. cloacae* in this study were resistant to ampicillin (100%), amoxicillin–clavulanate (100%), cephalothin (100%), cefroxime (100%), and cefoxitin (97.56%) (Fig. 3). Amikacin was highly effective against ESBL-*E. cloacae* (100%), followed by piperacillin-tazobactam, imipenem, meropenem, and levofloxacin (96.34%) (Fig. 3). Moreover, 76% to 92% of the *E. cloacae* strains were susceptible to cefepime, cefotaxime, ceftriaxone, ciprofloxacin, gentamicin, trimethoprim-sulfamethoxazole, levofloxacin, tigecycline, nitrofurantoin, and aztreonam. During our investigation, we observed an increase in antimicrobial resistance among strains of *E. cloacae* to the use of traditional antibiotics, which highlights the need for surveillance programs to monitor antimicrobial resistance.

Most of the ESBL-*E. cloacae* strains were resistant to at least three of the drugs tested. Table 3 shows the calculation of the MAR index for the 82 ESBL-*E cloacae* isolates in UTIs. Overall, ESBL-*E. cloacae* strains had an average MAR index of 0.37. Based on MAR scores of 0.67, the majority of *E. cloacae* isolates showed multidrug resistance to AMP, AMC, USAN, CXM, CAZ, FOX, FEP, CTX, CIP, GEN, SXT, TZP, IPM, TGC, and ATM. In contrast, *E. cloacae* strains had the lowest MAR index of 0.19. There were several strains with a MAR index exceeding 0.20. Consequently, most ESBL-*E. cloacae* strains were highly resistant to a wide range of antimicrobial drugs commonly used to treat UTIs, leading to high MAR index values.

**Table 3:** Multiple antibiotic resistance (MAR) index of ESBL-*E. clacae* isolated from urine samples

No. of resistant antibiotics	Profile of antimicrobial resistance	MAR index
14	AMP, AMC, USAN, CXM, CAZ, FOX, FEP, CTX, CIP, GEN, SXT, TZP, IPM, TGC, and ATM	0.67
12	AMP, AMC, USAN, CXM, FOX, CTX, CRO, SXT, FEP, TGC, NIT, and GEN	0.57
8	AMP, AMC, USAN,CXM, FOX, CIP, SXT, and NIT	0.38
7	FOX, CIP, SXT, GEN, SXT, TZP, and NIT	0.33
6	ATM, TGC, TZP, CXM, FOX, CIP, SXT,	0.29
4	MEM, ETP, MEM, and FEP	0.19
3	CTX, CXM, and TGC	0.14
Total average		0.37

## Discussion

Today, with the rise of antibiotic resistance, it is crucial to have a thorough understanding of microbial ecology (Andersson et al. 2020; Selvarajan et al. 2022). In recent years, the environment has been identified as both an incubator and a means of resistance propagation. This includes considering both environmental bacteria and the various types of microorganisms present in the human body (Martinez 2009; De Florio et al. 2018). Due to the selection pressure exerted in different environments, microorganisms are adapting and evolving resistance rapidly (Roemhild et al. 2018; Nguyen et al. 2020). In the healthcare setting, microorganisms can quickly develop and acquire mobile genetic components that are resistant to antibiotics (Ibrahim 2023; Mancuso et al. 2023a; Salam et al. 2023). This can lead to the spread of dangerous illnesses through their propagation.

Among the *Enterobacter* species, *E. cloacae* is most commonly isolated from healthcare settings (Bennett et al. 2023; Haque et al. 2023). Identifying *Enterobacter* species can be challenging because multiple species have very similar phenotypical characteristics. Additionally, as a member of the ESKAPE group, *Enterobacter* has been found to be resistant to various antibiotic classes (Mulani et al. 2019). *E. cloacae*, one of the 22 recognized *Enterobacter* species, has been most frequently identified and investigated in hospitalized patients. According to Jain and his colleagues' findings in 2021, *E. cloacae* was the most commonly isolated species, accounting for over 85% of the cases, followed by *E. aerogenes* and *E. asburiae* (Jain et al. 2021). The majority of the samples from which *Enterobacter* species have been isolated (over 50%) are urine and wound samples. Therefore, it is crucial to use a rapid and accurate technology that can precisely identify and differentiate *E. cloacae* from other types of *Enterobacter* species (De Florio et al. 2018). With this in mind, our investigation focused on using MALDI-TOF mass spectrometry technology to identify *E. cloacae* at the species level.

Based on our results, MALDI TOF MS was capable of identifying 185 out of 189 (97.88%) *E. cloacae* strains isolated from UTI patients. The mass spectrum provided by the MBT Compass Software was sufficient to identify almost all tested strains of *E. cloacae* at the species level. To improve the accuracy of our investigation, upgrading the Compass software could have been beneficial. De Florio et al. (2018) obtained similar results when they tested the effectiveness of MALDI platforms in identifying and clustering *Enterobacter* species from nosocomial environments. Their study found that MALDI TOF MS was a highly sensitive and specific technique for identifying and grouping *Enterobacter* species.

A study conducted by Godmer et al. (2021) utilized MALDI-TOF MS technology and two databases to identify isolates of the *E. cloacae* complex, instead of using the *hsp60* gene sequencing technique for identification. The study's results significantly improved the identification of members of the *E. cloacae* complex, with only 8% of the isolates misdiagnosed at the species level. Previously, *Enterobacter* isolates were detected using the MBT and Vitek MS, which have recently been adopted by medical microbiological laboratories due to their speed and affordability (Angeletti 2017). Both MALDI-TOF systems were used simultaneously to identify the *E. cloacae* complex, and their performance was evaluated. Except for *Enterobacter asburiae* identification, which was only accurate using the MBT, both devices demonstrated high sensitivity and specificity.

The MALDI-TOF MS technology has benefits and drawbacks, just like any other microbial identification method (Elbehiry et al. 2016, 2022a; Haider et al. 2023). This method can detect a wide range of microorganisms, including Gram-positive and Gram-negative bacteria, as well as fungi (Biswas and Rolain 2013). However, the presence of endospores from bacteria like *Bacillus* species can cause spectrum disruption. To resolve this issue, it is recommended to use a 24-h culture (Clark et al. 2013). MALDI-TOF MS can identify anaerobic isolates at the species level in hospital samples, but it cannot recognize Gram-positive isolates at the species level. To address this limitation, the reference library should be updated to include reference spectra from this bacterial group in the future (Chalupová et al. 2014). MALDI-TOF has been used to assess several clinical specimens, such as milk, urine, and blood, and it has been shown to accurately detect bacteria (Greub and Prod’Hom 2011; Wilson et al. 2019). However, blood and urine samples contain a large number of bacteria, which makes this method ineffective. To overcome this barrier, various technologies are available, such as urine filtering and blood separation (Tsuchida et al. 2020).

There is a global problem associated with the emergence and spread of Enterobacteriaceae that are resistant to multiple drugs, especially those that produce ESBLs (Saravanan et al. 2018; Teklu et al. 2019; Mustafai et al. 2023). The emergence of healthcare-associated infection outbreaks caused by ESBL-producing *E. cloacae* and *E. coli* has been reported in numerous cases (Rordorf et al. 2000; Kun Chen et al. 2021). The ESBL-*E. cloacae* pathogen has been recognized for some time as one of the predominant pathogens causing both nosocomial and public outbreaks worldwide (De Florio et al. 2018; Jain et al. 2021; Msimango et al. 2023). The severity of these outbreaks differs by region (Shakya et al. 2017; Saipriya et al. 2018). Understanding the geographic demographics and how they have changed over time is essential for determining the appropriate first-line antibiotic therapy for different regions. Several factors, such as healthcare practices, regulations, and the healthcare system, are intertwined and contribute to a significant burden on our community as a whole (Kettani Halabi et al. 2021; Abalkhail et al. 2022).

The findings of our study show that all *E. cloacae* isolates displayed a high degree of resistance to ampicillin, amoxicillin–clavulanate, cephalothin, cefuroxime, and cefoxitin. Similar findings were reported by Stock et al. (2001), who explained that *Enterobacter* species are naturally resistant to penicillin, amoxicillin–clavulanate, first-generation cephalosporins, and cefoxitin due to their production of *AmpC*-lactamase. In a recent investigation conducted by Jain et al. (2021), *E. cloacae* demonstrated 100% resistance to ampicillin and amoxicillin-clavulanic acid. Their study found that the frequency of ESBL-*Enterobacter* species decreased from 35% in 2017 to approximately 15% in 2020. However, beta-lactam resistance remained constant, illustrating the existence of developed mechanisms of resistance. Another challenge is that *E. cloacae* is capable of adapting to the healthcare environment, acquiring multiple genomic elements, and modulating the production of different proteins associated with antibiotic resistance. These factors complicate the treatment of infections caused by this bacterium (Intra et al. 2023).

Based on the observed tolerance to beta-lactam and cephalosporin antibiotics, these medications may no longer be suitable for use as therapeutic treatments (Abalkhail et al. 2022). However, our investigation indicates that most of *E. cloacae* isolates were susceptible to carbapenems (meropenem, ertapenem and imipenem), aminoglycosides (amikacin and gentamycin), and fluoroquinolones (ciprofloxacin and levofloxacin). Therefore, these antimicrobial agents are suggested as suitable treatments for UTIs. The results of our study agree with those of Jimenez-Guerra et al. (2020), who determined that the vulnerability of *E. cloacae* strains to carbapenem (imipenem) was greater than 80%. Matsumoto and Muratani (2004), for example, claim that imipenem has excellent antibacterial activity against fresh urine isolates, such as *E. cloacae*. However, another study concluded that only 20% were susceptible to imipenem (Dehkordi et al. 2022). This bacterium is an opportunistic, multi-resistant pathogen capable of causing infection outbreaks at hospitals (Davin-Regli and Pagès, 2015).

Additionally, increasing the broad use of these antibiotics may help alleviate concerns about future resistance development rates (Abalkhail and Elbehiry 2022). As this matter illustrates, antibiotic resistance in uropathogenic microorganisms is a serious global health problem. A study by Hryniewicz et al. (2001) has shown that bacteria in UTIs are increasingly resistant to traditional pharmaceuticals. The susceptibility of *E. cloacae* strains associated with UTIs to antibiotics has significantly declined over the past few years due to epidemiological surveillance. It seems likely that the results were caused by bacteria acquiring and transmitting resistance genes via plasmids through conjugation and transformation (Dehkordi et al. 2022). Additional variables that could have contributed to significantly enhanced resistance in *E. cloacae* strains would include self-treatment and non-compliance with dosage (Dehkordi et al. 2022).

The economic impact of infections triggered by multidrug-resistant bacteria can be difficult to assess due to varying costs in different healthcare systems (Huebner et al. 2016; Giraldi et al. 2019; Abalkhail and Elbehiry 2022). While calculating expenditures is challenging, it is likely that this phenomenon has adverse financial effects. These effects include extended hospital stays, increased medical expenses, higher laboratory and testing costs, the need for additional measures, greater expenditures for implementing mitigation and prevention applications, and potential legal action (Giraldi et al. 2019). Given the ongoing changes in healthcare, advancements in technology, legislative systems, and the need to maximize healthcare funds, it is crucial to recognize the financial implications of illnesses produced by multidrug-resistant bacteria (Elbehiry et al. 2019a, 2022d; Bassetti and Giacobbe 2020). This understanding will enable the implementation of affordable and effective control and prevention programs. Analyzing the social and economic implications of hospital infections is increasingly important for the development of prevention and surveillance systems. The involvement of practitioners from various fields is necessary to implement effective intervention strategies (Abalkhail et al. 2022; Elbehiry et al. 2022c).

Although there are several antimicrobial therapies available for treating *Enterobacter* diseases, the resistance to these treatments makes these bacteria more dangerous and likely to spread (Davin-Regli and Pagès, 2015). However, no vaccination has been developed yet to prevent these bacterial infections. Therefore, there is an urgent need for an effective vaccine to combat these infections. In 2022, Naveed and his colleagues conducted a trial using immunoinformatic methodologies to develop a safe, engineered, and efficient vaccine (Naveed et al. 2022b). They studied four *E. cloacae* proteins to identify epitopes for the development of an mRNA vaccine that would protect against infection with *E. cloacae*. Immunoinformatics methods were utilized to construct the vaccine in order to stimulate cellular and humoral reactivity. Several in vitro studies have demonstrated that *Enterobacter* is associated with epigenetic changes throughout infections (Davin-Regli and Pagès, 2015; Cairns et al. 2021). These epigenome regulatory mechanisms allow host organisms to prosper (Naveed et al. 2021, 2022a). A vaccine or treatment for this infection has been developed to address this function. Based on this theory, an increase in indigenous immune cells is a result of an increase in epigenetic modifications that affect both the immune system’s genes and “trained immunity” (Naveed et al. 2022c).

This study is likely to have a noteworthy influence on the controlling of UTIs in Riyadh, Saudi Arabia. However, there are several limitations to our study, particularly the fact that the research sample is limited to a specific Saudi state. According to the study, antibiotic resistance may not be widespread in other parts of the kingdom since the research was conducted only in Riyadh. To gather more information, it would be beneficial to identify a larger number of patients from different regions of Saudi Arabia who have the same medical condition. One of the challenges is the delay in administering appropriate medication due to the time required for urine testing and notification of ESBL UTIs. Severe cases of UTIs can lead to complications such as kidney damage and hypertension, as well as hinder treatment and diagnosis. Furthermore, conducting genomic identification and detailed genealogical research may offer additional insights into the distribution of ESBL-producing *E. cloacae* strains.

In conclusion, MALDI-TOF MS is a robust and precise mass spectrometry technology used to detect and differentiate *E. cloacae* in urine from patients with UTIs. This technique has multiple potential applications, including the recognition, discrimination, and determination of antimicrobial resistance in various clinical and environmental samples. It is important to note that *E. cloacae* isolates have shown increasing resistance to various antibiotics, such as ampicillin, amoxicillin–clavulanate, cephalothin, cefuroxime, and cefoxitin, with the potential for future carbapenem resistance. Using antibiotics for UTIs caused by ESBL-*E. cloacae* may be unnecessary, as it increases the spread of bacteria resistant to antibiotics. Additionally, multidrug-resistant bacteria can develop as a result of incorrect prescription drug use. Consequently, treating infections caused by this bacterium may become challenging, posing a significant risk to human health. Therefore, there is an urgent need to develop a highly effective vaccine to combat these bacterial infections.

There are certain limitations of the present study, including a relatively small sample size and the lack of validation in a larger cohort of patients. Additionally, the results obtained through MALDI-TOF MS were not validated using a molecular identification method, such as loop-mediated isothermal amplification or PCR. In the future, research on the diversity of the *K. pneumoniae* genome may reveal differences in virulence, epidemiology, evolution, and genetic characteristics. Through next-generation sequencing, we will be able to gain a better understanding of the molecular causes of infectious diseases such as *K. pneumoniae*. Sequencing the entire genome allows scientists to discover mutations associated with virulence, epidemiology, evolution, and genetic characteristics. This knowledge can be used to develop more effective therapies and management techniques.

## Data Availability

All data produced or analysed during this study are included in this published article.
